# Simplifying Proton NMR Spectra by Instant Homonuclear Broadband Decoupling^**^

**DOI:** 10.1002/anie.201300129

**Published:** 2013-06-03

**Authors:** N Helge Meyer, Klaus Zangger

**Affiliations:** Institut für Chemie/Organische und Bioorganische Chemie, Karl-Franzens Universität GrazHeinrichstrasse 28, 8010 Graz (Austria)

**Keywords:** homonuclear decoupling, NMR spectroscopy, pure shift, structure elucidation, TOCSY

NMR spectroscopy is a versatile tool to determine structural, chemical, and physical properties of molecules. One-dimensional ^1^H NMR spectra probably represent the most often acquired type of NMR data. However, compared to other NMR detectable nuclei, ^1^H spectra typically suffer from low resolution and severe signal overlap, mainly arising from extensive scalar coupling between protons. Homonuclear broadband decoupling, which leads to a collapse of ^1^H signals into singlets, has been suggested to be a solution to overcome the problem of poor signal dispersion in ^1^H NMR spectroscopy. Indeed, it was recently shown that the gain in resolution obtained in ^1^H spectra by broadband proton decoupling is comparable with the theoretical signal dispersion of regular spectra measured at several GHz.[1]

Over the last 30 years several methods have been developed which achieve homonuclear broadband decoupling.[2–14] However, these techniques have not been widely used, since they are insensitive and usually complicated processing schemes have to be applied to achieve the desired homonuclear decoupling. Recently, the slice-selective broadband-decoupling during a weak gradient field (also called Zangger–Sterk or ZS method)[14] has been rediscovered and improved to become a more general way to record pure shift spectra.[1, 7, 15]

The ZS-method employs the concept of slice-selective excitation by a weak magnetic gradient field to decouple all spins: Different parts of the NMR sample experience different magnetic field strengths when a linear gradient is applied. Therefore, a location-dependent frequency shift Δ*ω* = γ*G*s across the sample volume over a length of *s* is established, with the gyromagnetic ratio of the observed nucleus *γ* and the gradient strength *G*. While the gradient is active, a selective pulse thus excites the whole spectrum. However, different signals are excited in different parts of the sample, so that a spatially resolved excitation is achieved (Figure 1). Selective decoupling is implemented by a combination of a soft and a hard 180° pulse applied during a gradient of the same strength. Consequently, all signals that are off-resonance are inverted and the observed on-resonance signals remain unaffected.

**Figure 1:**
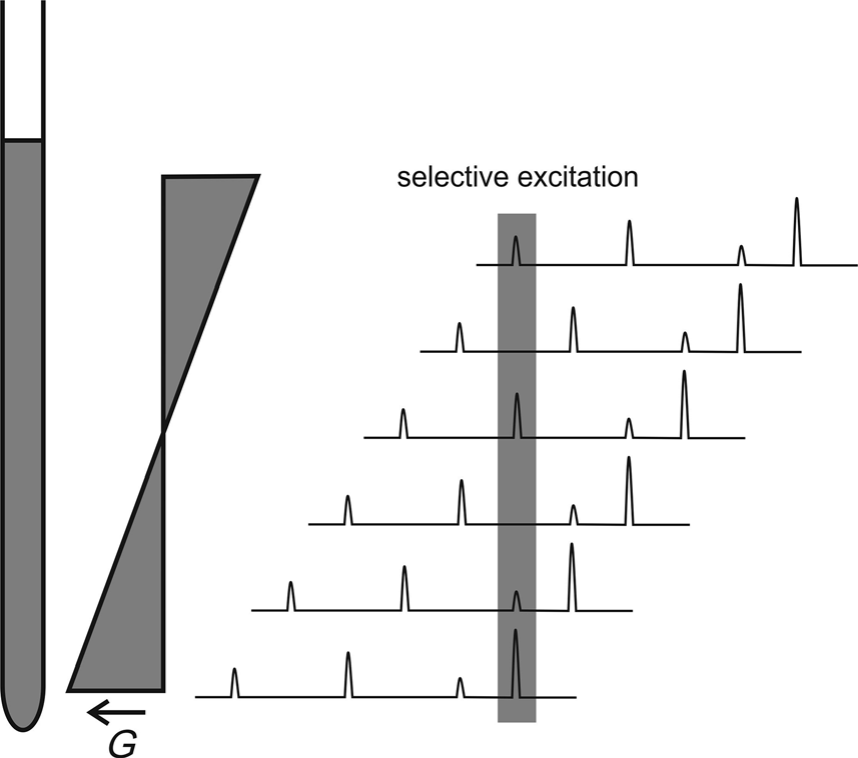
Selective excitation during a weak field gradient leads to an excitation of the whole spectrum in which each signal is irradiated in a different slice of the sample volume.

In a zeroth-order average Hamiltonian approximation, decoupling at a time *τ* is achieved if the coupled spin is inverted at *τ*/2. Since scalar coupling evolves slowly compared to chemical shift, a free induction decay of usually 10–20 ms in length, referred to as “data chunk”, can be recorded without intrusion of significant coupling effects. A full free induction decay (FID) is recorded in a pseudo 2D fashion by incrementing the delay *τ* between excitation and acquisition and concatenating all recorded chunks to a full FID of the desired length.[14]

This homonuclear decoupling scheme comes along with two severe drawbacks: Low sensitivity owing to the slice (spatially) selective excitation and, probably even more troublesome, the need to acquire a series of data chunks, which in turn also requires a special processing scheme to link them together. The sensitivity drops by a factor of Δ*ω*/(γ*G*s), with the excitation bandwidth of the selected pulse Δ*ω* (Hz), the gradient strength *G* (*G* cm^−1^), and the total length of the detected sample volume *s* (cm). Typically, sensitivity in a ZS-decoupled experiment is only a few percent of that attained in a non-decoupled spectrum. This decrease in sensitivity is partially compensated by the collapse of multiplets into single lines and also becomes less severe considering recent advances in spectrometer hardware, in particular cryogenic probes. However, even more detrimental to its general use is that each FID is composed of several data chunks, usually around 30, each recorded in an individual spectrum. In an additional processing step, the FID has to be reconstructed from these incremented FIDs in a pseudo 2D experiment. The necessity to acquire a series of spectra to obtain one decoupled 1D spectrum leads to a further significant reduction in sensitivity per measurement time.

Another promising technique is the BIRD-based homonuclear decoupling. Bilinear rotation decoupling (BIRD) pulses are able to distinguish protons, which are bound to ^13^C from those which are bound to ^12^C. In the BIRD-based homonuclear decoupling experiment only a subset of protons, namely those bound to ^13^C are excited and can be decoupled from protons bound to ^12^C by a BIRD pulse.[5] This experiment suffers from low sensitivity, since the natural abundance of carbon is around 1.1%. Thus, under ideal conditions, BIRD-decoupled spectra can reach only 1.1% of the sensitivity of a regular spectrum. In contrast, ZS-based decoupling allows for optimization of the signal-to-noise ratio by reducing the gradient strength. In addition, BIRD decoupling is somewhat less flexible than the ZS-based decoupling since it is restricted to carbon-bound protons and it cannot be used on uniformly ^13^C-labeled compounds. A major advantage of the BIRD-decoupling, however, is the possibility to decouple strongly coupled protons, this cannot be readily achieved with the ZS method.[15] In this regard ZS- and BIRD decoupling can be used to gather complementary information. Very recently, Lupulsecu et al. described the use of BIRD pulse trains during individual data acquisition blocks to achieve single-shot broadband decoupling of 1D ^1^H spectra and avoiding additional processing schemes.[16]

Herein, we present a general approach to achieve pure-shift spectra which completely removes any sophisticated processing and greatly reduces acquisition time by using ZS-based homonuclear decoupling sequences. This improvement is achieved by ZS-type homonuclear broadband decoupling during acquisition, that is, in a single scan decoupling sequence, comparable to the BIRD single scans. These instant homo-decoupled spectra can be recorded like regular 1D spectra, or be used in the acquisition dimension of multidimensional spectra, without any special processing scripts and no additional “decoupling dimension”. Through this reduced dimensionality the method also provides huge sensitivity gains per measurement time over conventional pure-shift methods.

To decouple the direct dimension we interrupt the acquisition approximately every 1/3(^3^*J*_HH_) to send in the ZS-decoupling block. It is critical that any chemical shift evolution during the decoupling is strictly avoided, so that the FID is not distorted. One particularly convenient side effect of the ZS decoupling is the fact that during the weak gradient field all detected signals are “on resonance” and therefore do not experience chemical shift evolution. Any delays necessary outside the gradient, for example, for gradient recovery, are placed symmetrically around the two 180° pulses so that the chemical shift is refocused. Morris and co-workers proposed improvements for the ZS decoupling experiment.[1] One of these is the timing of the decoupling pulse in such a way, that decoupling of the signals is centered in the middle of the data chunk, so that each piece of the FID can, in principal, be twice as long. To achieve the same effect, the first acquisition block is only half as long as the next one (Figure 2). While the acquisition is interrupted for decoupling, the magnetization is relaxing. Therefore, it is critical to keep the pulses for decoupling as short as possible to limit the effect of relaxation during decoupling. Otherwise, the FID will become discontinuous, which leads to artifacts in the spectrum after Fourier transformation. However, the selective pulse has to be long enough to allow for decoupling of close signals. That is why there is a trade-off between clean, highly resolved spectra and the possibility of decoupling two signals with very similar resonance frequencies. As a compromise we found that a 10 ms Gaussian pulse, corresponding to a theoretical excitation bandwidth of approximately 88 Hz leads to nicely decoupled spectra with artifacts arising only for very close resonances. As an example, a comparison of a conventional and an instant homo-decoupled spectrum of the macrolide antibiotic azithromycin,[17, 18] is shown in Figure 3.

**Figure 2:**
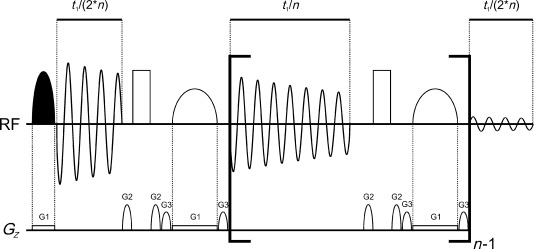
Pulse sequence used to record instant homo-decoupled spectra. Slice-selective excitation is accomplished by a band-selective 90° pulse applied during a weak field gradient. Decoupling is achieved by a combination of a hard 180° pulse and band selective 180° pulses applied during a gradient of the same strength as used during excitation applied approximately every 1/3(^3^*J*_HH_). Acquisition and decoupling is alternated *n* times. Note, that the first fraction of acquisition is only half as long as the subsequent ones. Thereby, full scalar decoupling is achieved in the middle of each fraction of the FID. Open rectangles represent hard 180° pulses. Solid and open semi-ellipses in RF represent soft 90° and 180° pulses, respectively.

**Figure 3:**
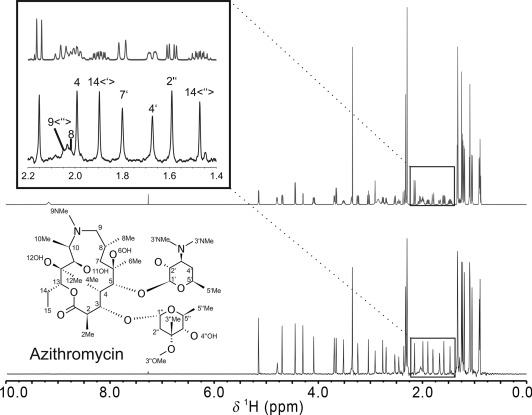
Regular ^1^H spectrum of 4 mm azithromycin in CDCl_3_ (top) and instant homo-decoupled version (bottom). Only the strongly coupled protons of the methylene group 8 and 9 cannot be resolved by this method. The instant homo-decoupled spectrum was recorded with 512 scans in 20 min. It would take almost 10 h to record a comparable spectrum with conventional decoupling schemes (e.g. pseudo2D ZS decoupling).

Any overlap in the crowded region between *δ* = 1.4 and 2.2 ppm is completely resolved using instant ZS-decoupling. Note, that the strongly coupled protons 8 and 9′′ cannot be decoupled with this experiment, because homonuclear decoupling is limited to weakly coupled spins. Pure shift spectra with instant homo-decoupling can be recorded much faster than with standard ZS decoupling. For example, a decoupled spectrum of 40 mm azithromycin can be recorded in a few seconds (Supporting Information Figure S2). Compared to the commonly used pseudo 2D approach, which reconstructs an FID from data chunks of 10–20 ms length, instant homo-decoupling can decrease the time to record pure shift spectra by a factor of 25–50 for an acquisition time of 500 ms.

Instant homo-decoupling can be readily implemented in the acquisition dimension of homonuclear 2D spectra, like, for example, NOESY or TOCSY experiments. While a number of decoupled 2D spectra have been reported, the decoupling was always effective in the indirect dimension, which typically shows much lower resolution. Decoupling of the direct dimension could only be achieved by an additional decoupling dimension and subsequent transfer of this information into the direct dimension. With our decoupling scheme we can directly decouple the acquisition dimension, resulting in very high resolution 2D spectra. Figure 4 and Figure S1 show a directly decoupled TOCSY spectrum of azithromycin, recorded with a large number of scans and increments in the indirect dimension to gain optimal resolution and signal-to-noise ratio. To demonstrate the capabilities of this method in routine applications Figure S2 shows an instant homo-decoupled TOCSY spectrum recorded with a shorter acquisition time (2.5 h).

**Figure 4:**
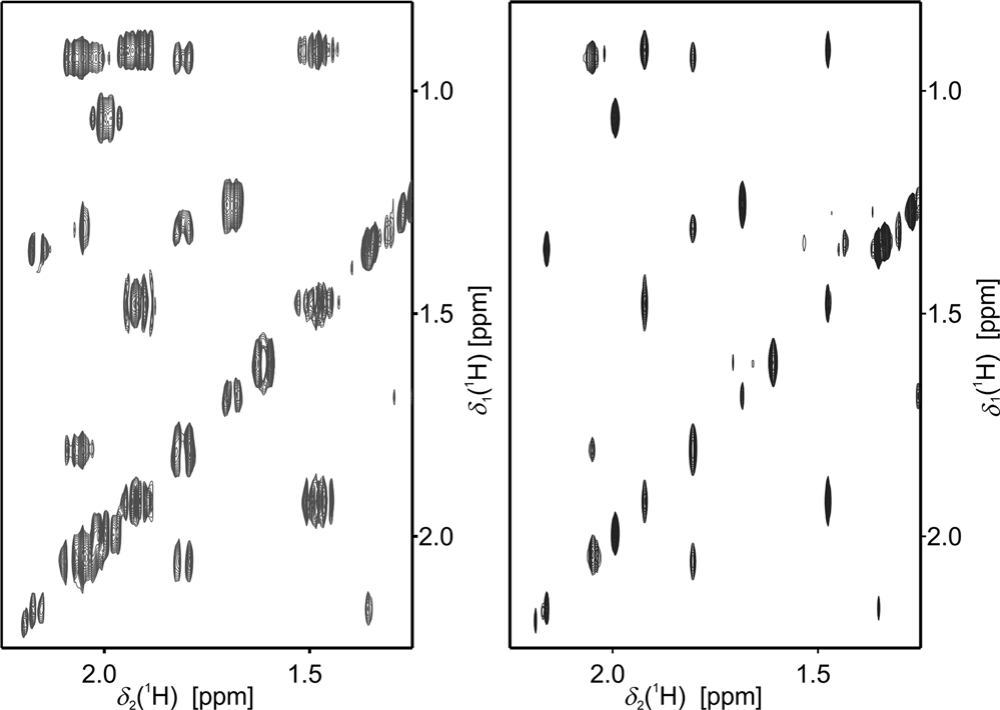
Expanded view of a regular TOCSY of 40 mm azithromycin in CDCl_3_ (left) and an *ω*_2_-decoupled one (right). Spectra were acquired with a mixing time of 60 ms and 256 increments in the indirect dimension. Total acquisition times were approximately 2 h for the regular TOCSY (8 scans per increment) and approximately 20 h for the homonuclear decoupled TOCSY (64 scans per increment).

To test whether this method is also applicable to bigger, faster relaxing molecules, we measured decoupled spectra of CM15, a synthetic antimicrobial peptide which has been characterized by NMR spectroscopy.[19, 20] Typically, spectra of even small peptides show severe signal overlap. For example, it is not possible to identify individual signals of CM15 alpha protons. However, decoupling resolves signal overlap in this region (Figure S3). Also homonuclear 2D spectra, for example, a TOCSY spectrum (Figure S4), show much less signal overlap, so that resonance assignment is greatly simplified. Since ZS decoupling does not change the relative integral of a signal, even pure-shift NOESY spectra can be recorded and analyzed. A much larger number of distance restraints could be extracted from a decoupled NOESY spectrum, because signal overlap is greatly reduced. This is critical in the process of 3D structure determination by NMR spectroscopy.

In conclusion, we present a general approach to obtain pure-shift spectra which does not require any special data processing and provides huge gains in sensitivity per time compared to regular ZS decoupling. This method can be easily implemented in the acquisition dimension of standard 2D and 3D homonuclear pulse sequences, for example, TOCSY and NOESY spectra. Instant homo-decoupled 2D spectra of a sample at mm concentration can be recorded in minutes, rather than the hours needed for conventional ZS decoupling.

## Experimental Section

Experiments were acquired on a Bruker Avance III 500 MHz spectrometer at 298 K. 1D ^1^H NMR spectra were recorded using the pulse sequence in Figure 2. The same pulse scheme was implemented into a standard TOCSY sequence by replacing the last hard 90° pulse by a soft pulse applied during a weak field gradient. If not stated otherwise, we used a 60 ms Eburp[21] and a 10 ms Gauss pulse, respectively, under a slice selective gradient (G1) of 0.5 Gauss cm^−1^ to achieve spatially selective excitation and refocusing. To reduce artifacts we used sine shaped gradients with 4 and 5.5 Gauss cm^−1^ for G2 and G3. Spectra were recorded on 4 mm and 40 mm azithromycin in CDCl_3_.

## Notes

As a service to our authors and readers, this journal provides supporting information supplied by the authors. Such materials are peer reviewed and may be re-organized for online delivery, but are not copy-edited or typeset. Technical support issues arising from supporting information (other than missing files) should be addressed to the authors.
