# Supplementary material for: Simplifying Proton NMR Spectra by Instant Homonuclear Broadband Decoupling
Source: Angew Chem Int Ed Engl. 2013 Jun 3;52(28):7143–6. doi: 10.1002/anie.201300129 (PMC3790959; doi:10.1002/anie.201300129)
Supplement: Supplementary file 1 [file anie0052-7143-sd1.pdf]

Angewandte Chemie
Eine Zeitschrift der Gesellschaft Deutscher Chemiker

Supporting Information
© Wiley-VCH 2013
69451 Weinheim, Germany

Simplifying Proton NMR Spectra by Instant Homonuclear Broadband Decoupling**

*N. Helge Meyer and Klaus Zangger**

anie_201300129_sm_miscellaneous_information.pdf

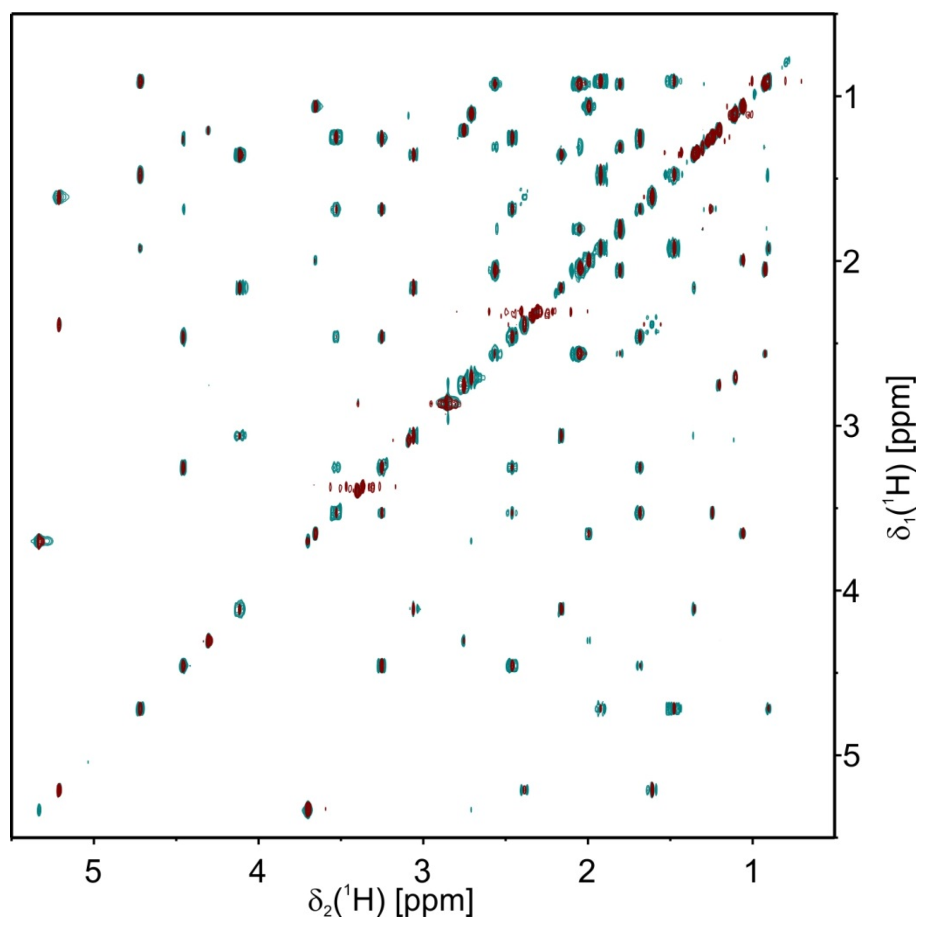

Supplementary figure 1:
Overlay of a regular (cyan) and an instant homodecoupled (red) TOCSY spectrum of 40 mM azithromycin in $CDCl_3$. Spectra were acquired with a mixing time of 60 ms and 256 increments in the indirect dimension. Total acquisition times were ~2 h for the regular TOCSY (8 scans) and ~20 h for the homonuclear decoupled TOCSY (64 scans).

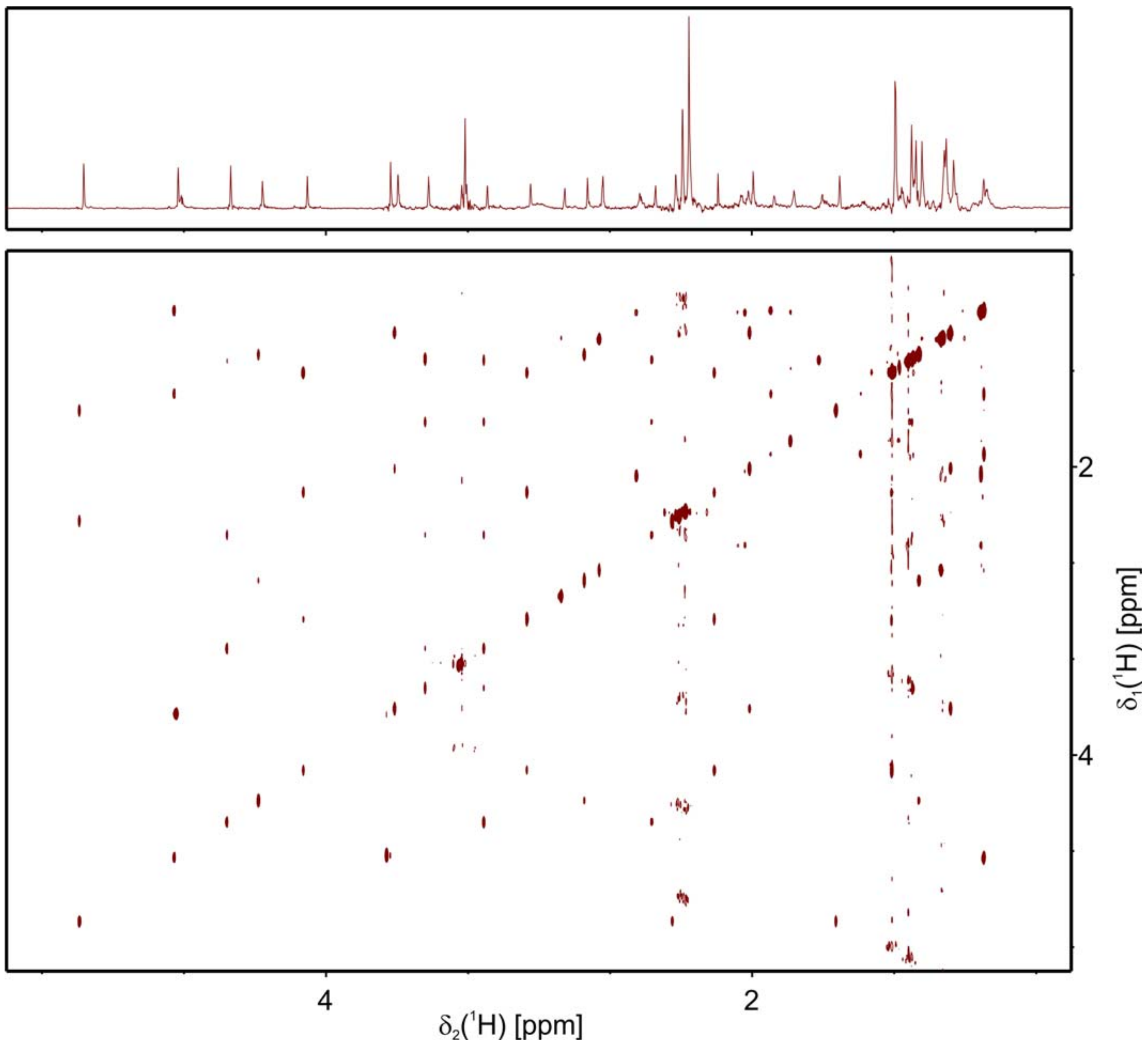

Supplementary figure 2:
Instant homodecoupled 1D ^{1}H (top) and TOCSY spectrum of 40 mM azithromycin in $CDCl_3$. A 30 ms Eburp and a 5 ms Gauss pulse under a slice selective gradient (G1) of 0.5 Gauss/cm were used for slice selective excitation and refocusing, respectively. 1D ^{1}H was recorded in 3 seconds (2 scans). TOCSY was recorded with a mixing time of 60 ms and 128 increments in the indirect dimension for a total acquisition time of ~2,5 h (16 scans).

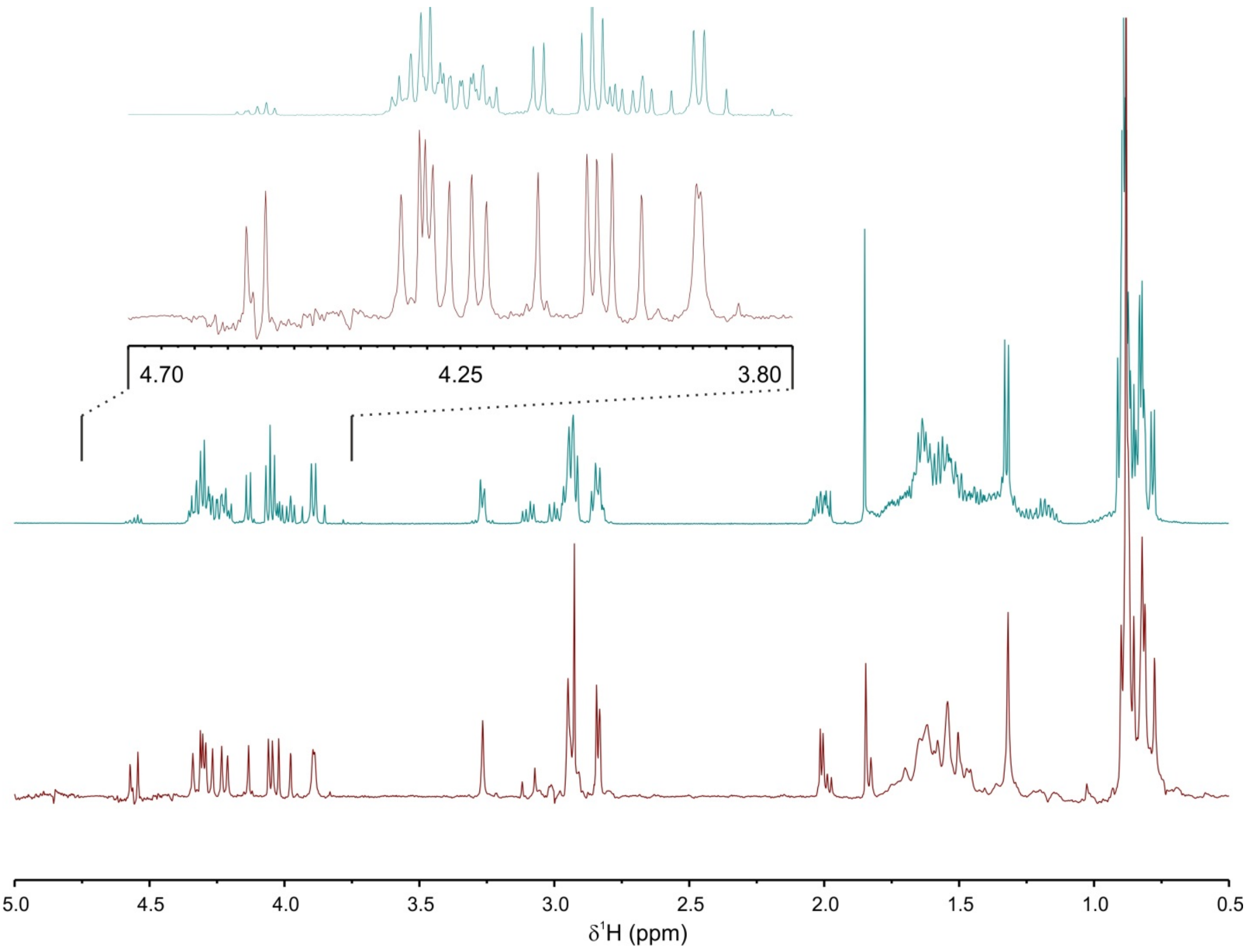

Supplementary figure 3:
Regular (cyan) and instant homodecoupled 1D ^{1}H spectrum of 2 mM CM15 peptide in D_2O. Each spectrum was acquired in ~20 min (512 scans). All $^{\alpha}$H protons are resolved in the pure shift spectrum. A 25 ms Eburp and a 4.5 ms Gauss pulse under a slice selective gradient (G1) of 0.5 Gauss/cm were used for slice selective excitation and refocusing, respectively.

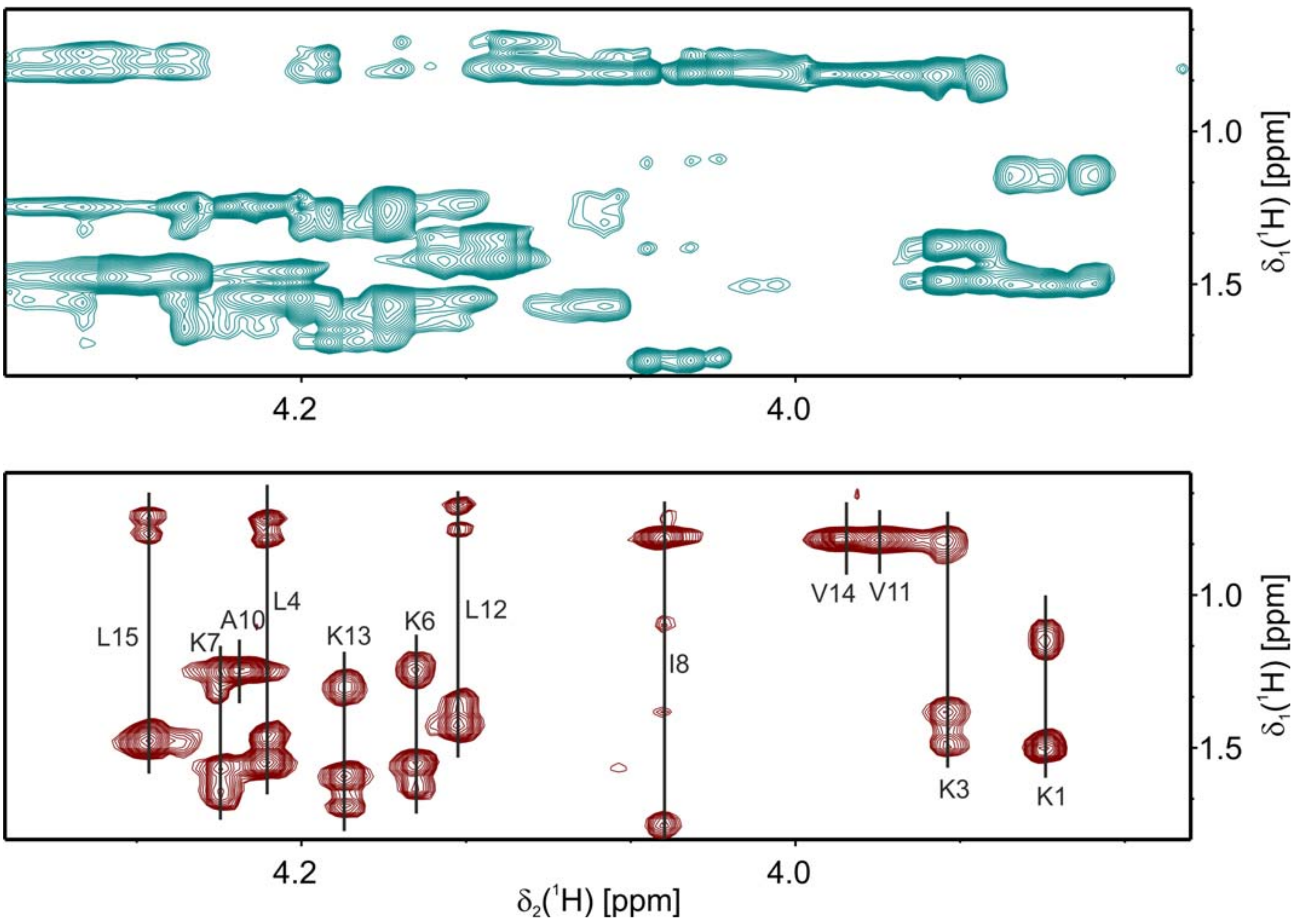

Supplementary figure 4:
$^{\alpha}H$ to aliphatic region of a TOCSY spectrum of 2 mM CM15 peptide in D_2O. Overlap in the pure shift TOCSY (red, decoupled in the direct dimension with instant homodecoupling scheme) is significantly reduced as compared to a regular TOCSY (green). Signals in this most crowded region of the TOCSY spectrum could not be assigned in the regular TOCSY but very easily in the homonuclear decoupled version. This assignment is indicated by the amino acid single letter code and residue number of CM15. Spectra were recorded with a mixing time of 60 ms and 128 increments in the indirect dimension. Sixteen scans were acquired for the regular TOCSY and 256 for the homonuclear decoupled TOCSY. A 25ms Eburp and a 4.5ms Gauss pulse under a slice selective gradient (G1) of 0.5 Gauss/cm were used for slice selective excitation and refocusing, respectively.
